# Changes to Yucatán Peninsula precipitation associated with salinity and temperature extremes of the Caribbean Sea during the Maya civilization collapse

**DOI:** 10.1038/s41598-017-15942-0

**Published:** 2017-11-20

**Authors:** Henry C. Wu, Thomas Felis, Denis Scholz, Cyril Giry, Martin Kölling, Klaus P. Jochum, Sander R. Scheffers

**Affiliations:** 1 MARUM - Center for Marine Environmental Sciences, University of Bremen, 28359 Bremen, Germany; 2 Present Address: Leibniz Centre for Tropical Marine Research (ZMT), 28359 Bremen, Germany; 3 Institute for Geosciences, Johannes Gutenberg University Mainz, 55099 Mainz, Germany; 4 Climate Geochemistry Department, Max Planck Institute for Chemistry, 55128 Mainz, Germany; 5 Marine Ecology Research Centre, Southern Cross University, Lismore, NSW 2480 Australia; 6 School of Earth and Environmental Sciences, The University of Queensland, St. Lucia, QLD 4072 Australia

## Abstract

Explanations of the Classic Maya civilization demise on the Yucatán Peninsula during the Terminal Classic Period (TCP; ~CE 750–1050) are controversial. Multiyear droughts are one likely cause, but the role of the Caribbean Sea, the dominant moisture source for Mesoamerica, remains largely unknown. Here we present bimonthly-resolved snapshots of reconstructed sea surface temperature (SST) and salinity (SSS) variability in the southern Caribbean from precisely dated fossil corals. The results indicate pronounced interannual to decadal SST and SSS variability during the TCP, which may be temporally coherent to precipitation anomalies on the Yucatán. Our results are best explained by changed Caribbean SST gradients affecting the Caribbean low-level atmospheric jet with consequences for Mesoamerican precipitation, which are possibly linked to changes in Atlantic Meridional Overturning Circulation strength. Our findings provide a new perspective on the anomalous hydrological changes during the TCP that complement the oft-suggested southward displacement of the Intertropical Convergence Zone. We advocate for a strong role of Caribbean SST and SSS condition changes and related ocean-atmosphere interactions that notably influenced the propagation and transport of precipitation to the Yucatán Peninsula during the TCP.

## Introduction

Numerous Classic Maya population centres and political systems on the Yucatán Peninsula disintegrated during the Terminal Classic Period (TCP; CE 750–1050)^1,2^. Spatially and temporally varying palaeoclimate evidence suggests significant changes in precipitation and hydrology from interannual^2,3^ to multidecadal^3–8^ timescales during the TCP. However, even with the accumulating climate evidence, researchers are reluctant to assign droughts as the main culprit of demise^1,9^ due to the oversimplification of various interacting factors. What researchers do sufficiently agree on are the complexities and multi-causality aspects of disintegration ranging from differences in geological and land-use features to social dynamics^9–11^. In addition, the potential to accelerate civil unrest and human conflict due to changes in climate^12^ may further amplify degradations in agriculture production, productivity, and population decline feedback^9,10^. Nevertheless, the strong interrelationship evidence between climate records and Maya settlement abandonment and political disintegration^2,12^ suggests a very powerful connection.

**Figure 1:**
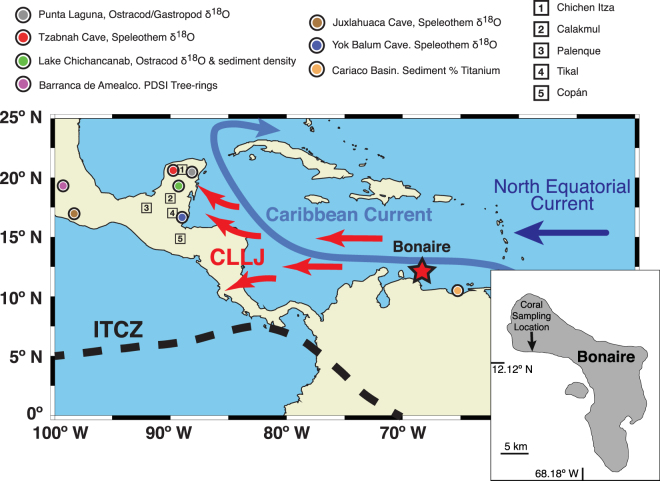
Research area and Bonaire fossil coral sampling location. Regional map representing the major atmospheric (Caribbean Low Level Jet; CLLJ; red arrows)^24,55^ and oceanic conditions (Caribbean Current and North Equatorial Current; light blue and dark blue, respectively) affecting Bonaire, Netherlands Antilles (12.5°N, 68.5°W; star). The approximate location of modern mean annual Intertropical Convergence Zone (ITCZ) is shown as black dashed line. The round symbols in Mesoamerica are terrestrial summer precipitation reconstruction locations, which observed noteworthy drought events and anomalous rainfall conditions during the Terminal Classic Period (TCP). The symbols of the terrestrial precipitation reconstructions are shown with its respective colours in Fig. 2 and S1: Punta Laguna, ostracod and gastropod δ^18^O^13^ (gray); Tzabnah Cave, speleothem δ^18^O^6^ (red); Lake Chichancanab, ostracod δ^18^O^4^ (green); Lake Chichancanab, sediment density^14^ (green); Barranca de Amealco, Palmer Drought Severity Index (PDSI)^3^ (pink); Juxlahuaca Cave, speleothem δ^18^O^8^ (brown); Yok Balum Cave, Belize speleothem δ^18^O^2^ (blue); Cariaco Basin, tuned sediment titanium %^2^ (orange). Square symbols distinguish the five major Maya archaeological sites or prominent Maya cities during the TCP. The inset map (digitally modified and produced from Google Earth base image, map data: SIO, NOAA, U.S. Navy, NGA, GEBCO; https://www.google.com/earth/) shows fossil coral collection location on the island of Bonaire, Netherlands Antilles (12.13° N, 68.23° W), in the Caribbean Sea. Regional map created using the base layer of Ocean Data View ver. 3^89^ (https://odv.awi.de) and modified manually.

**Figure 2:**
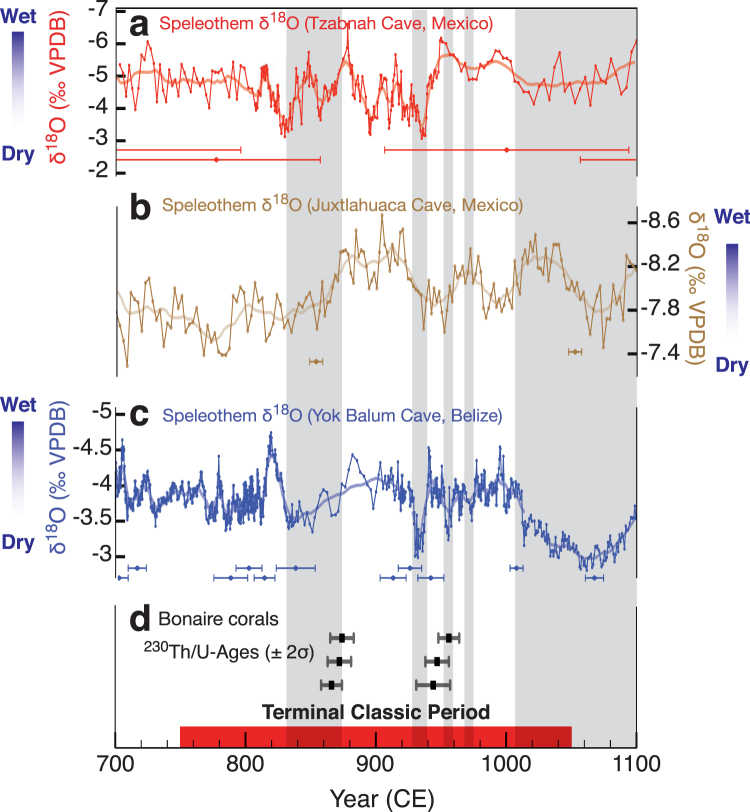
Bonaire fossil coral age and Yucatán Peninsula precipitation reconstructions. High-resolution (≤5 years) ^230^Th/U-age dated terrestrial summer precipitation records from Mesoamerica indicating differing drought conditions during the Terminal Classic Period (TCP; ~CE 750–1050). The records are ordered geographically from the north to the south: (**a**) Tzabnah Cave, Mexico (speleothem δ^18^O)^6^, (**b**) Juxlahuaca Cave, Mexico (speleothem δ^18^O)^8^, and (**c**) Yok Balum Cave, Belize (speleothem δ^18^O)^2^. The horizontal error bars below the individual published records in panels **a-c** indicate the respective ^230^Th/U-age dating uncertainty of each record. (**d**) The ^230^Th/U-ages (±2σ age error) of the *Orbicella annularis* sensu lato (*Orbicella annularis* ‘species complex’) coral colonies in this study. The shaded bars demarcate the drier-than-average periods adopted from the Yok Balum, Belize stalagmite record^2^.

Current discussions surrounding potential climatic influence during the TCP invoke a southward displacement of the summer position of the Intertropical Convergence Zone (ITCZ)^5^ resulting in pronounced drier conditions across the Maya lowlands^2,5^. However, the mounting evidence of TCP climate change is exclusively based on terrestrial proxy records of summer precipitation^2–8,13,14^, which in part show obvious differences and are often attributed to dating uncertainties and regional climate variability^1–3,6–8^ (Figs 1 and 2, and S1). Still ambiguous for the TCP is the influence of the tropical Caribbean Sea, which is known to be an important mediator of Central American and Caribbean climate on seasonal and interannual to decadal timescales^15–18^. Understanding the changes in Caribbean sea surface conditions during the TCP on these timescales of relevance to the Maya collapse is particularly important because it is the main atmospheric moisture supply of Central America^15^. Clearly, palaeoceanographic records resolving interannual to decadal sea surface conditions in the Caribbean Sea during the TCP are essential to enhance our understanding of the underlying mechanisms that resulted in the hydrological changes over Yucatán Peninsula during this key time period in human history.

Here we present snapshots of reconstructed variability in sea surface conditions during the TCP from bimonthly-resolved δ^18^O and Sr/Ca records of southern Caribbean fossil corals from Bonaire. The climate of Bonaire is influenced by the trade winds and the westward flowing Caribbean Current that is a part of the northward-flowing surface conduit of the Atlantic Meridional Overturning Circulation (AMOC)^19^. Modelling studies identified the southern Caribbean Sea as a region of strong SST and SSS response in simulations of AMOC slowdown on millennial timescales^20,21^. Precipitation at Bonaire is characterized by the main rainy season from October to January and a midsummer ‘drought’^22,23^. This indicates that Bonaire is not influenced by the seasonally migrating ITCZ, because the northernmost ITCZ position that is reached during boreal summer is located south of Bonaire. In contrast, precipitation in the Caribbean Sea and Mesoamerica has been shown to be primarily driven by the changing strength of the Caribbean Low Level Jet (CLLJ)^17,22,24–26^. The CLLJ is a maximum easterly zonal wind (greater than 13 m s^−1^) observed in the lower troposphere (~925 hPa) as an extension of the Atlantic trade winds propagating atmospheric moisture across this region (Fig. 1).

Modern calibration studies of the Caribbean and Atlantic coral *Orbicella annularis* sensu lato (*Orbicella annularis* ‘species complex’) have demonstrated the applicability of the Sr/Ca and δ^18^O values to record sea surface temperature (SST) variability^27–30^. Because coral δ^18^O reflects a combination of SST and the ambient δ^18^O of seawater (δ^18^O_sw_), the combination of both δ^18^O and Sr/Ca facilitate the reconstruction of δ^18^O_sw_ by removal of the coevolving temperature component^31,32^. Moreover, the variability of δ^18^O_sw_ has been shown to be linearly related to sea surface salinity (SSS) in the Caribbean^33^. Thus, our results derived from fossil corals of Bonaire can be used to discuss the crucial role of large-scale ocean circulation and Caribbean Sea ocean-atmospheric interaction processes in modulating moisture transport and hydrological cycle changes of Mesoamerica during the TCP. As evidence indicating dry conditions on the Maya Lowlands of Mesoamerica continues to build, ocean circulation and ocean-atmospheric interaction processes have often been overlooked that likely complement the often-suggested southward displacement of the ITCZ^2,5^.

## Results

Our reconstruction of southern Caribbean Sea surface conditions variability is based on the analysis of coral cores collected at Bonaire (12°N, 68°W; Fig. 1). Fossil coral skeletal preservation analyses by x-radiograph imaging (Fig. S2), powder x-ray diffraction (XRD) analysis (Table 1), and thin-section microscopy (Fig. S3) are prerequisites for palaeoclimate reconstructions with fossil corals from any region^34–37^ and indicate excellent aragonite preservation. XRD results are all well within the limit of minor alteration in fossil corals for palaeoclimate studies from the undetectable to less than 2% calcite (Table 1). Verification of the fossil corals’ suitability for palaeoclimate research by representative thin-section analyses indicate excellent preservation of primary porosity, clear dissepiments, and well-defined centres of calcification with no evidence of secondary aragonite or calcite cements near regions of micro-sampling (Figs S2 and S3).

**Table 1:** Bonaire coral analytical results.

Coral Sample ID	^230^Th/U Age (CE)	Internal Years	Mean Annual Extension Rate (mm)	Mean δ^18^O (‰VPDB)	Mean Sr/Ca (mmol mol^−1^)	Sampling Path Calcite Content (%)
BON-1-BI	**865** ± **8**	71	9.3 ± 2.6	−3.48 ± 0.20	9.29 ± 0.08	≤2
BON-3-F-C	**872** ± **9**	27	12.4 ± 4.4	−3.27 ± 0.41	9.30 ± 0.09	0
BON-1-AII	**874** ± **8**	41	9.6 ± 3.1	−3.69 ± 0.23	9.25 ± 0.07	0
BON-3-DII	**944** ± **13**	57	12.0 ± 3.6	−3.46 ± 0.28	9.33 ± 0.10	0
BON-3-B	**947** ± **9**	36	11.6 ± 2.4	−3.10 ± 0.36	9.37 ± 0.07	≤1
BON-3-F-B	**956** ± **8**	32	9.1 ± 2.4	−3.57 ± 0.33	9.35 ± 0.12	≤1
BON-9-E	**1945** ± **7**	42	6.2 ± 1.5	−4.15 ± 0.23	9.24 ± 0.08	0

Bonaire coral ages determined by ^230^Th/U-dating in CE with 2σ-uncertainty (Table 2) are ordered from oldest (top) to youngest (bottom). The internal years of each individual colony are determined from the combination of annual cycles in Sr/Ca and δ^18^O measurements in addition to verification by x-radiographs of skeletal density banding (Fig. S2). Mean annual extension rate (mm), δ^18^O (‰ VPDB), and Sr/Ca (mmol mol^−1^) of each individual coral time-series are listed with the 1σ standard deviation. The per cent calcite content (%) in the vicinity of the carbonate micro-sampling path of each individual fossil coral is measured by x-ray diffraction (XRD).

**Figure 3:**
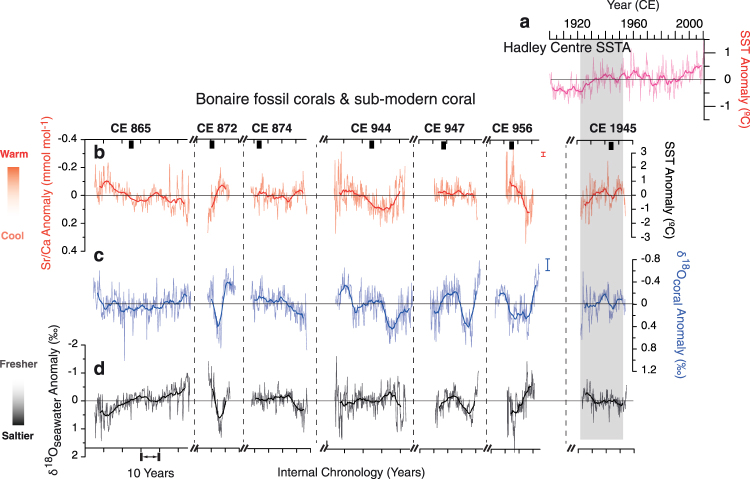
Bimonthly Sr/Ca and δ^18^O records of Bonaire *Orbicella* corals and reconstructed δ^18^O_sw_. Bonaire fossil coral records for the TCP and one sub-modern record (comparison as modern baseline condition) with the corresponding ^230^Th/U-ages shown above each coral colony. (**a**) Hadley Centre Sea Surface Temperature Anomaly for the Bonaire grid (11.5°N, 67.5°W)^40^. (**b**) Coral Sr/Ca anomaly records (analytical uncertainty =  ± 0.019 mmol mol^−1^ (1σ)) and Sr/Ca-derived SST anomalies using the *Orbicella* spp. coral Sr/Ca-SST relationship of −0.092 mmol mol^−1^ per 1 °C^30^. The sub-modern coral Sr/Ca-derived SST anomaly record over the period 1923–1965 is presented as modern baseline condition that tracked the interannual variability of the Hadley Centre Sea Surface Temperature Anomaly for the Bonaire grid. This proxy to instrumental coherence shown under the shaded bar verified the SST reconstruction methodology for the TCP corals.(**c**) δ^18^O anomaly values (analytical uncertainty =  ± 0.07‰ VPDB (1σ)). (**d**) The reconstructed δ^18^O_sw_ values as a proxy of SSS. The lack of reliable instrumental SSS records (Fig. S7) prohibited calibration and verification of the SSS-δ^18^O_sw_ relationship for proper scaling. All coral geochemical records are reported as departures from the bimonthly mean for the entirety of each coral to facilitate comparison because of inter-colony offsets. To highlight interannual and longer-term lower frequency variability, we applied a 5- year smoothing to the records (bold lines).

The fossil coral ^230^Th/U-ages with corresponding 2σ-uncertainty range from CE 865 ± 8 years to CE 956 ± 8 years (Table 2) places the entire coral collection precisely in the TCP (Fig. 2 and S1). Due to inter-colony offsets in mean Sr/Ca and δ^18^O (Table 1), all bimonthly-resolved coral records are reported as anomalies (departures from the mean monthly climatology) over each individual record to facilitate comparison (Fig. 3). The mean propagated Sr/Ca-SST reconstruction (relative SST) uncertainty of all individual corals is ±1.0 °C (1σ) incorporating the analytical and Sr/Ca-SST calibration errors^38^ (Fig. S4). The propagated uncertainty of reconstructed δ^18^O_sw_ (±0.16‰; 1σ)^38^ is smaller compared to published studies from the Caribbean^39^ (Fig. S4). Furthermore, bimonthly-resolved records (Sr/Ca, δ^18^O, and δ^18^O_sw_) from Bonaire fossil corals reveal significant variance at interannual timescales and enhanced variance at decadal timescales (Figs S4 and S5).

**Table 2:** Bonaire coral ^230^Th/U-ages.

Coral ID	^238^U (µg/g)	^232^Th (ng/g)	(^230^Th/^238^U)	(^234^U/^238^U)	(^234^U/^238^U) _initial_	Age corrected (before 2012)	Age (CE)
BON-1-BI	2.30 ± 0.01	0.0297 ± 0.0005	0.01199 ± 0.00008	1.148 ± 0.002	1.148 ± 0.002	1146.4 ± 8.3	**865** ± **8**
BON-3-F-C	2.30 ± 0.01	0.0577 ± 0.0007	0.01192 ± 0.00009	1.148 ± 0.002	1.149 ± 0.002	1139.8 ± 9.1	**872** ± **9**
BON-1-AII	2.22 ± 0.01	0.0359 ± 0.0005	0.01192 ± 0.00008	1.150 ± 0.003	1.151 ± 0.003	1137.7 ± 8.3	**874** ± **8**
BON-3-DII	2.70 ± 0.01	0.079 ± 0.001	0.0112 ± 0.0001	1.150 ± 0.002	1.150 ± 0.002	1068.1 ± 12.6	**944** ± **13**
BON-3-B	2.57 ± 0.01	0.0730 ± 0.0009	0.01117 ± 0.00009	1.151 ± 0.003	1.151 ± 0.003	1065.3 ± 9.0	**947** ± **9**
BON-3-F-B	2.44 ± 0.01	0.0360 ± 0.0006	0.01105 ± 0.00008	1.147 ± 0.003	1.148 ± 0.003	1056.3 ± 8.5	**956** ± **8**
BON-9-E	2.303 ± 0.002	0.0048 ± 0.0001	0.00066 ± 0.00007	1.148 ± 0.003	1.148 ± 0.003	67.0 ± 7.4	**1945** ± **7**

Results of ^230^Th/U-dating of the Bonaire coral colonies. All ages are presented as both **age corrected** (years before 2012) and **age** (CE) with the corresponding 2σ-uncertainty.

Based on the location of the ^230^Th/U-dating sampling (Fig. S2) and our internal chronology calculations (Table 1), possible overlapping time windows exist between the fossil coral collection. Two possible minor overlapping time windows exist with either 2–3 or 6–7 years of overlap when the ages are set at the extremes of the 2σ ^230^Th/U-dating uncertainty. This thus only accounts for < 2–8% of overlap when merged (Fig. S6). It is therefore inappropriate to splice together these very short overlapping time windows at either annual or interannual resolution with insignificant overlap within the 2σ-age uncertainties. However, when shifted towards the other end of the extremes of the 2σ-age uncertainties, we acknowledge the lack of flawless ‘match’ between individual proxy records (Fig. S6). Coral vital or calcification effects can be ruled out for the apparently imperfect ‘splicing’ of these records because the corresponding δ^13^C and growth rate records do not show suspicious shifts or jumps (not shown), and the growth rates of all TCP colonies of >9 mm per year are relatively high (Table 1). Due to these limitations, we consider the presentation as individual coral time windows and not as spliced records to be the most accurate representation of our results that minimizes the inherent age-errors.

**Figure 4:**
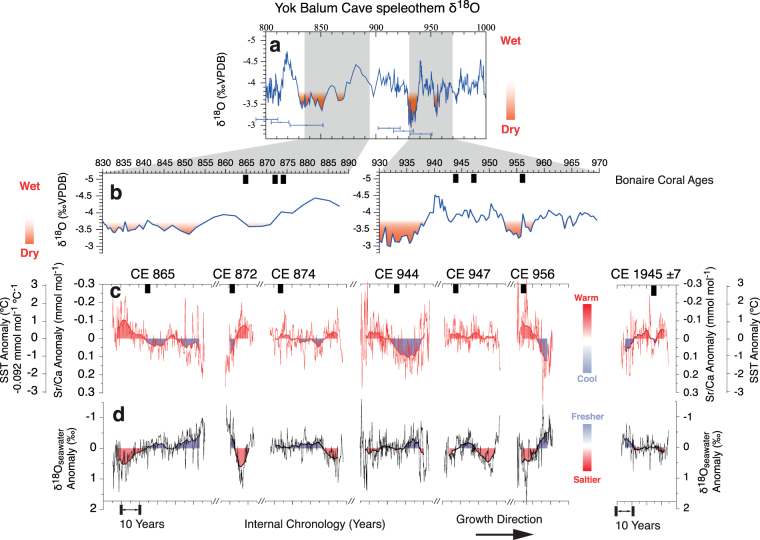
Comparison of Bonaire coral-derived Sr/Ca-SST and reconstructed δ^18^O_sw_ records to the Yok Balum Cave, Belize speleothem record. Six Bonaire fossil corals that indicate large perturbations in SST and SSS are shown (**a**) with the most well-dated Yok Balum Cave speleothem δ^18^O record^2^ (precipitation) throughout the entire TCP. The horizontal error bars indicate the dating uncertainties of this summer precipitation record. The precision coral ^230^Th/U-age is shown (black squares) along with (**b**) close-up overlapping time windows of the speleothem δ^18^O record^2^. (**c**) The Sr/Ca-derived SST anomaly and (**d**) reconstructed δ^18^O_sw_ anomaly (proxy of SSS) records during the TCP (similar to Fig. 3). The lack of reliable instrumental SSS records (Fig. S7) prohibited calibration and verification of the SSS-δ^18^O_sw_ relationship for proper scaling. The fossil coral records depict warm/saltier (orange) and cool/fresher (blue) conditions similar to the drought conditions (orange shadings) of the speleothem record.

Over a recent 42-year period, CE 1923–1965, the interannual to decadal variations in the Hadley Centre SST anomaly record^40^ for the 1° by 1° grid over Bonaire (11.5°N, 67.5°W) agree well with the Sr/Ca-derived SST anomaly record overlapping the sub-modern coral (CE 1945 ± 7 years; Fig. 3). Even though the full magnitude of the sub-modern coral-derived SST anomaly slightly overestimates the instrumental record based on the previously published calibrations, the close agreement between the sub-modern coral and gridded instrumental SST anomaly records indicates that the reconstruction of relative SST changes on interannual to decadal timescales during the TCP is possible from our fossil Bonaire corals using the sub-modern coral as benchmark. We note that the maximum magnitude of interannual to decadal variability in the instrumental SST anomaly record^40^ is ±0.6 °C, which is less than the ±1.4 °C maximum interannual to decadal oscillation magnitude of the coral Sr/Ca-derived SST anomaly record of the entire fossil coral collection (Figs 3 and 4).

The inferred changes of SSS from the reconstructed δ^18^O_sw_ indicate pronounced high-amplitude interannual to decadal variability of SSS during the TCP that are concomitant to those of SST (Fig. 4). This interannual to decadal-scale change of 0.63‰ during the TCP is twice the magnitude of the subtle variability from sub-modern coral condition (0.32‰). Based on modern calibrations of northern Caribbean *Orbicella* spp. linear SSS-δ^18^O_sw_ relationship of 0.20‰ per psu^33^, the interannual to decadal SSS variability during the TCP is appreciably elevated by at least 1.5 psu compared to the early twentieth century (Figs 3 and 4). Unfortunately, continuous and replicable modern instrumental SSS datasets going back to the 1940s are not currently available^41,42^ for verification against our sub-modern coral-based reconstruction (Fig. S7). Differences between individual gridded instrumental datasets (Fig. S7) can produce a difference of up to 1.3 psu (e.g. September 1989) equating to a 0.26‰ change in Caribbean *Orbicella* spp. coral reconstructed δ^18^O_sw_ based on *Orbicella* sp. SSS-δ^18^O_sw_ relationship^33^ hindering our ability for a thorough calibration and validation. These larger instrumental differences than our propagated δ^18^O_sw_ uncertainty of the Bonaire fossil corals (±0.25‰)(Figs 3 and 4) validates the ability of corals to accurately record the possible magnitude and range of local SSS change. The pronounced oscillations in δ^18^O_sw_ and intense decadal-scale climate trends during the TCP that are significantly larger than the modern baseline record exemplifies the peculiar climate differences from ~1100 years ago. Moreover, the reconstructed δ^18^O_sw_ variability around CE 872 and CE 956 is also the largest observed since the mid-Holocene^43^ showing the intriguing perturbations of climate during the TCP.

## Discussion

Our most striking find is that the Bonaire coral records indicate a stronger interannual to decadal variability of SST and especially SSS in the southern Caribbean Sea during the TCP compared to the modern baseline (Figs 3 and 4). Importantly, the coral records of ~CE 865, ~CE 872, and ~CE 956 indicate periods of significant anomalously high SST and SSS on these timescales that may be considered as temporally coherent to the TCP precipitation anomalies (droughts and decreased rainfall) in the Yucatán documented by a Belize speleothem record^2^ (Fig. 4). These periods of SSS extremes on interannual to decadal timescales are accompanied by anomalously high SST (corals ~CE 865, ~CE 872, and ~CE 956). Moreover, instances of significant coherence (r = 0.70–0.72; P < 0.001) are observed with long-term secular freshening trends in the southern Caribbean (~CE 865 and ~CE 956) corresponding to increasing precipitation in Belize following the drought periods of ~CE 850 and ~CE 955 (Fig. 4).

Importantly, the pronounced interannual to decadal δ^18^O_sw_, therefore SSS, perturbations in the southern Caribbean Sea during the TCP indicated by the coral records cannot be explained by local ITCZ-related precipitation changes because Cariaco Basin sediments suggest that the mean northernmost position of the summer ITCZ during the TCP was similar to today^5,44^, i.e., located south of Bonaire over the South American continent. This is consistent with coral evidence suggesting that ITCZ-related summer precipitation at Bonaire has not occurred since the mid-Holocene^43^. Furthermore, we can exclude an advected ITCZ-related summer precipitation signal from the Orinoco River as the Orinoco plume exhibits a maximum trajectory towards the northern Caribbean^45,46^ spreading minimally into our research area today. We therefore conclude that the pronounced changes in southern Caribbean SSS during the TCP are not the result of local summer precipitation changes. Alternatively, we suggest that large-scale ocean circulation processes may be the potential driver of the anomalously pronounced interannual to decadal SSS and concomitant SST variations in the southern Caribbean Sea during the TCP.

**Figure 5:**
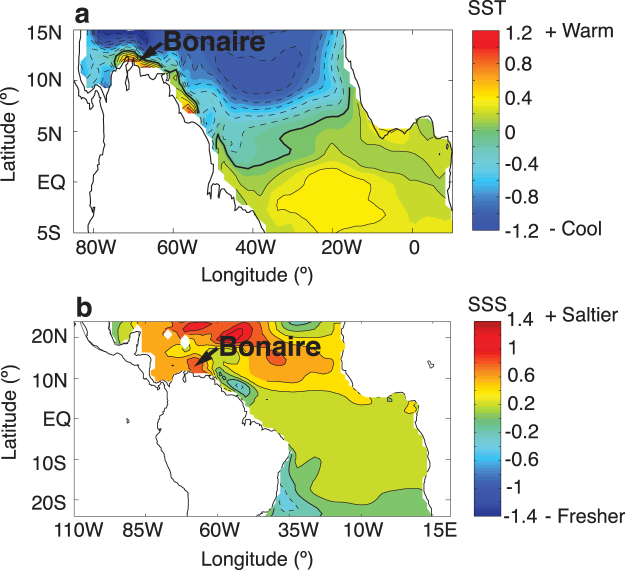
AMOC modelling results near Bonaire. Coupled ocean-atmosphere model simulation results of reduced Atlantic Meridional Overturning Circulation (AMOC) indicating variability of SST^20^ and SSS^21^. (**a**) The model simulation results of SST are modified from Fig. 2 of ref.^20^ showing the SST difference between the Combined-Forcing Experiment (surface-forcing/effect of atmospheric processes and boundary-forcing/effect of ocean circulation processes) and the Control Experiment over the tropical Atlantic. (**b**) Model simulation results of SSS modified from Fig. 3 of ref.^21^ showing the SSS difference between the Combined-Forcing Experiment (surface-forcing/effect of atmospheric processes and boundary-forcing/effect of ocean circulation processes) and the Control Experiment over the tropical Atlantic. Arrows indicate Bonaire location.

Modelling studies identified the southern Caribbean Sea as a region of strong SST and SSS response in simulations of AMOC slowdown on millennial timescales^20,21^. Interestingly, our reconstructed interannual to decadal anomalies of concomitant high SSS and high SST that are potentially linked to the Yucatán precipitation anomalies of the TCP have the same sign as the southern Caribbean SSS and SST response to a weakened AMOC in simulations mimicking the Younger Dryas event^20,21^ (Fig. 5). Although changes in AMOC strength over time has frequently been attributed in causing millennial-scale climate perturbations in the Atlantic region^47^, the behaviour of this large-scale ocean current system over interannual to decadal timescales remains uncertain^48^. We speculate that our reconstructed interannual to decadal periods of anomalously high SSS and high SST in the southern Caribbean are possibly due to a weakened AMOC on these timescales. The potential for decadal to multidecadal changes in AMOC strength is evident from modelling studies^48,49^ and modern observations^50,51^, providing support for our hypothesis of a potentially enhanced interannual to decadal variability of the AMOC during the TCP.

Interestingly, in the above mentioned model simulations^20,21^, AMOC weakening on millennial timescales results in a narrow strip of anomalously warmer surface water that appears off the northern coast of South America, extending into the Southern Caribbean (Fig. 5). This warming is surrounded by wide spread surface cooling of the North Atlantic, with the latter representing the well-known response in simulations of a weakened AMOC^20,21^. Importantly, the weakened AMOC induces strong meridional SST gradient anomalies in the Caribbean Sea with warmer SST at Bonaire and cooler SST in the north (Fig. 5)^20^. Modern Caribbean Sea SST anomalies^52^ also indicate a robust relationship to regional precipitation^53^ over the entire southern Caribbean Sea (Fig. S8). This contrast in Caribbean SST (strong meridional SST gradient) in turn leads to the changing strength of the CLLJ^24,54^. These geostrophic relationships of the ocean and atmosphere suggest an intense dynamic feedback, which reinforces the strength and condition of the CLLJ^24,55^.

An increased CLLJ strength can significantly impact the propagation of atmospheric moisture for rainfall over Mesoamerica and the greater Caribbean region by suppressing deep convection due to high vertical wind shear^24,55,56^. Moreover, modern reanalysis studies have linked anomalous Mesoamerican droughts throughout the last century to decreased tropical convection resulting from decreased easterly waves due to strengthened CLLJ^56^. These results indicate that the CLLJ is a major contributor to droughts and precipitation anomalies over the recent centuries^57^ by changes in the moisture flux divergence in the Caribbean. Changes in CLLJ strength over Bonaire and the Caribbean Sea from modern reanalysis also reveal synchronous significant coherence to precipitation on the Yucatán Peninsula (Fig. S8). The amount of precipitation recorded on the Yucatán Peninsula is directly related to the increase or decrease in east to west winds at Bonaire (Fig. S8). This process demonstrates the possible extremes in anomalous precipitation events during the TCP due to the movement of precipitation from the Caribbean Sea based on CLLJ strength (Fig. S8).

However, geographical differences contributing to anomalous precipitation occurrences in Mesoamerica can and do occur^55^. Observational studies of the CLLJ have shown both enhanced and decreased rainfall across Mesoamerica due to orographic enhancement with difference ranging from the north to the south and from the Pacific to the Caribbean coasts^55,58^. Importantly, these modern differences of the CLLJ can potentially explain the obvious differences in palaeo-observations^1–3,6–8^ during the TCP that has not been previously investigated. Therefore, it is apparent that not a single major drought event (or several drought events) impacted the entire Yucatán Peninsula at the same time but rather a series of high amplitude hydroclimatic changes on interannual to decadal timescales, which affected different areas of the Maya Lowlands in different ways. Thus, we suggest that the physical mechanism that connects the pronounced interannual to decadal SST and SSS variability in the southern Caribbean Sea with the Yucatán precipitation anomalies of the TCP involves changes in the meridional gradient of Caribbean SST leading to changes in the strength of the CLLJ that controls the propagation or suppression of atmospheric moisture towards the Yucatán Peninsula.

Our results may be reconciled with summer precipitation reconstruction studies that invoke the southward excursion of the ITCZ^2,5^ during the TCP, because one prominent outcome in modelling studies of AMOC slowdown is the anomalous southern position of the ITCZ^59,60^. We do not rule out the temporary southward excursion of the ITCZ over this region, but rather suggest that it cannot be the simple sole mechanism for explaining the observed hydrological anomalies of the Yucatán Peninsula during the TCP. For example, exacerbating the negative rainfall anomalies over Mesoamerica during the TCP may include the influence of El Niño Southern Oscillation (ENSO) warm events that are linked to times of stronger CLLJ^24^. Interestingly, fossil coral spectral analysis results indicate the presence of significant periods between 5.7–6.6 years in both Sr/Ca and δ^18^O variability (Fig. S5) with the 5.7 years period identified as the most prominent variability in the cospectrum of ENSO and the North Atlantic Oscillation (NAO)^61^. Thus, the combined ENSO-NAO interactions as indicated by the prominent 5–6 year oscillation^61^ in the Bonaire coral records suggest NAO-ENSO teleconnections were probably impacting SST and SSS variability in the Caribbean region during the TCP, similar to today^62^.

Our findings provide evidence for anomalously strong interannual to decadal SSS and SST variability in the Caribbean Sea during the TCP, a time interval known for the decline of the Classic Maya civilization on the Yucatán Peninsula. We find that extremes of SSS and SST in the southern Caribbean on these timescales can be considered to coincide with prominent precipitation anomaly events of the TCP in the Yucatán. The concomitant SSS and SST variations on interannual to decadal timescales reflect ocean circulation processes, which may be fundamentally linked to an enhanced variability of the AMOC at that time. We suggest that changes in the Caribbean meridional SST gradient and associated changes in the strength of the Caribbean low-level atmospheric jet that significantly controls the moisture transport from the Caribbean Sea to Mesoamerica^22,24^ provide a physical mechanism linking the anomalous interannual to decadal variability of SSS and SST in the southern Caribbean and of precipitation in the Yucatán during the TCP. Our results advocate for a strong role for regional ocean-atmosphere interactions, and provide a new perspective on the hydrological changes in the Yucatán during the TCP that is an addition to the oft-suggested southward displacement of the ITCZ^2,5^ at that time.

## Methods

### Coral Sampling and Background

Six large fossil coral boulders were cored from coastal deposits at the northwestern shore of Bonaire (Netherlands Antilles; 12.13°N, 68.23°W; Fig. 1). These coral boulders were most likely deposited by extreme wave events such as palaeo-tsunamis or severe storms^63,64^. Previous study discuss in detail the location and coral core extraction methods^36^. Additionally, one sub-modern coral colony (42 years of growth) was collected for calibration purposes. Bonaire is located ~100 km north of the Venezuelan coastline and ~400 km northwest of Cariaco Basin in the Caribbean Sea experiencing open-ocean conditions as part of the Leeward Antilles. A total of 5.1 meters of coral cores were retrieved and cut following previously detailed coral core extraction and slabbing methods^36,43^.

The coral colonies were determined to be *Orbicella annularis* sensu lato (*O. annularis* ‘species complex’) after examination of the outer corallite shape and growth architecture by x-radiographs (Fig. S2) and thin-sections (Fig. S3). The *O. annularis* ‘species complex’ representation contains the three cryptic Atlantic *Orbicella* species (*O. annularis sensu stricto*, *O. faveolata*, *O. franksi*). These Atlantic corals have recently been revised from the genus *Montastraea* to the genus *Orbicella* ^65^.

### Coral δ^18^O and Sr/Ca analyses

All Bonaire coral cores were prepared for micro-sampling and sampled under the same conditions. Coral powder samples were acquired sequentially at 1 mm intervals using a micro-drill with a 0.8 mm steel drill bit at ~1 mm depth along the fastest growing axis. The samples were analysed for stable isotope and Sr/Ca measurements following previously described methods^34–36,43^ with the same accuracy and precision. Homogenized skeletal powder samples were split into aliquots for δ^18^O and Sr/Ca analyses at MARUM-Center for Marine Environmental Sciences, University of Bremen, Germany following previously described methods^34–36,43,66^. Briefly, δ^18^O values were measured on a Finnigan MAT 251 mass spectrometer with replicate measurements of an internal carbonate standard (Solnhofen Limestone calibrated against NBS-19) better than ±0.07‰ VPDB (1σ)^66^. δ^18^O measurements for colonies age CE 1945 ± 7 years, CE 947 ± 9 years, and CE 944 ± 13 years were completed at 1 mm resolution. The colonies with age of CE 956 ± 8 years, CE 874 ± 8 years, CE 872 ± 9 years, and CE 865 ± 8 years were analysed at every-other mm. All measurements were completed to retain at a minimum of 6 samples per year between each density banding pairs.

Sr/Ca analyses for all coral colonies were analysed at every 1 mm resolution on an Agilent 720 series simultaneous axial inductively coupled plasma–optical emission spectrophotometer (ICP-OES) at MARUM. Repeated measurements of a laboratory coral standard with a set Ca concentration allowed for offline instrumental drift correction and yielded Sr/Ca ratios of 8.6–10 mmol mol^−1^. Relative standard deviation of the entire Sr/Ca analyses was better than 0.2%. 86 aliquots of a *Porites* coral powder reference material, JCp-1^67^ were analysed as samples with an average Sr/Ca of 8.916 ± 0.019 mmol mol^−1^ (1σ) obtained during the course of this study.

X-radiographs of the slabs reveal clear annual-density banding patterns, the growth geometry, and guided the micro-sampling tracks (Fig. S2). The internal age chronology of each individual coral colony was developed following a combination of the depths of annual skeletal density banding from the x-radiographs (Fig. S2) and timing of sub-seasonal δ^18^O and Sr/Ca variability. Since the TCP fossil corals’ internal ages span across a range (32 to 71 years) and contain an average time-window growth length of 44 years, this 42-year sub-modern coral is an adequate representative analogue snapshot of modern southern Caribbean Sea baseline conditions. This typical average 40-year window is also the commonly applied length using sub-modern corals for calibration to reconstruct climate from fossil corals^68,69^.

### Fossil coral skeletal preservation

Subsamples from every individual coral colony were removed close to the micro-sampling transects for fossil coral skeletal preservation analyses (Figs S2–S3). Obvious anomalous density patches on the top and bottom sections of each coral colony that are typical of diagenetic alterations were omitted in the micro-sampling procedure. Prerequisites for palaeoclimatic work on any fossil corals^34–37^ include the examination of coral skeletal material by x-radiograph imaging (Fig. S2), powder XRD analysis (Table 1), and thin-section microscopy (Fig. S3), showing good preservation. Powder XRD results in the vicinity of the sampling transects are all within the limit of minor alteration in fossil corals for palaeoclimate studies^34–37^ using previously described methods^70,71^ (Table 1). XRD analyses of the fossil corals revealed pristine or ‘excellent’ preservation with 100% aragonite content for four of the seven colonies (including the sub-modern colony). Two colonies contain calcite content that were ≤1% calcite. The colonies containing less than 1% calcite are considered to be “good” preservation. Calcite content at less than 2% was found in the oldest fossil colony and is considered as “fair” preservation.

Because the presence of secondary (inorganic) aragonite cannot be detected by XRD, petrographic thin-sections were produced. The thin-sections indicate excellent skeletal preservation of primary porosity, clear dissepiments, and well-defined centres of calcification^72^ with no evidence of secondary aragonite overgrowth and without obvious signs of calcite cements near regions of micro-sampling (Fig. S3). A few sections of Sr/Ca values were omitted in the final analysis, discussion, and reconstruction of δ^18^O_sw_ variability based on possible subtle subaerial diagenesis^73^ that could not be detected by our thorough examination. Observation of depleted Sr/Ca and enriched Mg/Ca values in the record indicate subtle calcite presence without any systematic impact on coral δ^18^O values^73,74^.

### ^230^Th/U-age determination

Bulk samples for ^230^Th/U-age determination were removed near the sampling transects (Fig. S2). Due to sample size requirement for analysis, the samples contained between 1 and less than 3 years of coral growth. The sample preparation and ^230^Th/U-age determination followed established methodology^75^. ^230^Th/U-dating for the TCP fossil colonies was performed by multi-collector inductively coupled plasma mass spectrometry (MC-ICPMS) at the Max Planck Institute for Chemistry, Mainz, Germany. Analytical MC-ICPMS procedures involve a standard-sample bracketing procedure to derive correction factors for mass fractionation and Faraday cup to ion counter gain^76,77^. A detailed description of the calibration of the utilized mixed ^233^U-^236^U-^229^Th spike is given elsewhere^78^. The sub-modern coral from the last century was dated by thermal ionization mass spectrometry (TIMS) ^230^Th/U-dating with a MAT 262 RPQ TIMS at the Heidelberg Academy of Sciences, Germany, using the double filament technique following previously described sample preparation and analytical procedures^79^.

All activity ratios were calculated using the decay constants of ref.^80^ and corrected for detrital Th assuming a bulk Earth ^232^Th/^238^U weight ratio of 3.8 for the detritus and ^230^Th, ^234^U and ^238^U in secular equilibrium. The initial (^234^U/^238^U) activity ratios of all samples are in agreement with the initial (^234^U/^238^U) activity ratio of the modern coral (Table 2). All criteria for the reliability of fossil coral ^230^Th/U-ages^71,81^ are fulfilled and all ages presented herein are considered reliable within their 2σ-error or 95% Confidence Interval (Table 2). Our ^230^Th/U-ages place the entire coral collection precisely in the TCP (Fig. 2 and S1–S2).

### Fossil coral chronology development

The internal age chronology of each individual coral colony was developed using a combination of the depths of annual skeletal density banding from the x-radiographs and the timing of sub-seasonal δ^18^O and Sr/Ca variability. We concluded that high-density bandings were secreted in the warmest times of each year (September/October) coinciding with minimums in sub-seasonal δ^18^O and Sr/Ca variability. Thus, annual maximum Sr/Ca was set to March (average month of lowest SST) and annual minimum Sr/Ca was set to September (average month of highest SST). Sub-seasonal age estimates were then linearly interpolated into 6-points per year (bimonthly resolution) based on the δ^18^O and Sr/Ca results using the ARAND software package^82^.

### Bonaire coral-derived SST and δ^18^O_sw_ calculations

Sr/Ca ratios in modern Caribbean and Atlantic *Orbicella* spp. corals have been shown to represent SST variability over multiple timescales without a salinity effect (Table S1). For the calibration of the Sr/Ca records to SST, we use the most conservative, growth-corrected value (−0.092 mmol mol^−1^ °C^−1^; ref.^30^) from a range of published Sr/Ca-SST relationships in literature (Figs 3 and 4; Fig. S4; Table S1). We chose this Sr/Ca-SST calibration slope after considering multiple calibration slopes and sensitivities with this type of coral (Fig. S4 and Table S1). The larger SST calibration slope is considered to be more robust and more appropriate for the examination of mean-state, interannual, and decadal variability in coral-based palaeoclimate studies^83–85^. As demonstrated by the range of published Sr/Ca-SST calibration relationships due to sampling resolution differences, the relative magnitude and scaling of both SST and δ^18^O_sw_ reconstructions are reshaped but the procedure does not change the overall reconstructed trends of each record (Fig. 3 and S4). The result ranges from the conservative ~1 °C^30^ to the inconceivable ~9 °C^86^ (Fig. S4).

The δ^18^O-SST transfer function of −0.22% °C^−1^ (ref.^29^) was chosen for this study because Atlantic and Caribbean *Orbicella* spp. studies sampled under various resolutions are able to achieve similar calibration results (Table S2). Furthermore, lower sampling resolution has been shown to be just as efficient as higher sampling resolutions with similar δ^18^O to SST calibration relationships^29,33^. A *Orbicella* spp. study^29^ also suggested that the strategy of using a lower sampling resolution for corals from regions with a restricted annual temperature range such as Bonaire is less likely to experience sampling resolution issues. Moreover, another Caribbean *Orbicella* spp. study has demonstrated that the annual averages of δ^18^O obtained from adjusting sampling resolution between six to forty samples per year remained the same for corals living in a restricted temperature range^33^.

We calculated the instantaneous bimonthly δ^18^O_sw_ values following accepted methods^31,32^. In principal, these two essentially identical δ^18^O_sw_ calculation methods require the transfer functions of both δ^18^O-SST (−0.22‰ °C^−1^; ref.^29^) and Sr/Ca-SST (−0.092 mmol mol^−1^ °C^−1^; ref.^30^). This enable straightforward reconstruction of δ^18^O_sw_ values using the combination of our fossil coral δ^18^O and Sr/Ca values^32,43,83^. Combined analytical uncertainties and compounding errors associated with the various Sr/Ca-SST transfer functions were calculated for both Sr/Ca-derived SST and the reconstructed δ^18^O_sw_^38^ shown as grey error envelops (Fig. S4). Mean propagated Sr/Ca-SST reconstruction uncertainty of all fossil corals is ±1.5 °C (1σ) and the propagated uncertainty of reconstructed δ^18^O_sw_ is ±0.25‰ (1σ) (Fig. S4). To be conservative and cautious with our δ^18^O_sw_ reconstructions, we limit our discussion of Caribbean TCP climate to interannual to decadal variability because the reliability of our δ^18^O_sw_ reconstruction is most sensitive and dependent on the choice of *Orbicella* spp. Sr/Ca-SST relationship. Portions of Sr/Ca record from the tops and bottoms of the corals were not used for palaeoclimate reconstruction due to subtle subaerial diagenesis that did not impact δ^18^O values^73,74^.

Due to inter-colony offsets and to facilitate comparison, we report coral geochemical records as anomalies based on the departures from the bimonthly mean for the entirety of each individual coral. To highlight the interannual and longer-term lower frequency variability, we applied a 5-year smoothing to the coral δ^18^O, Sr/Ca, and δ^18^O_sw_ records (Figs 3 and 4; Fig. S4). Multi-Taper Method (MTM) spectral analysis^87^ was also completed for all records. The significance was determined relative to a red noise null hypothesis determined with the robust method of noise background estimation^88^ with mean seasonal cycles removed to estimate the power spectrum of each record (tapers of 3 and resolution of 2; Fig. S5).

### Data deposition

The Bonaire fossil coral data reported in this paper has been deposited at the information system PANGAEA (Data Publisher for Earth and Environmental Science; https://doi.pangaea.de/10.1594/PANGAEA.829390).

## Electronic supplementary material

Supplementary Information
