# Supplementary material for: Changes to Yucatán Peninsula precipitation associated with salinity and temperature extremes of the Caribbean Sea during the Maya civilization collapse
Source: Sci Rep. 2017 Nov 20;7:15825. doi: 10.1038/s41598-017-15942-0 (PMC5696513; doi:10.1038/s41598-017-15942-0)
Supplement: Supplementary file 1 — Supplementary Information [file 41598_2017_15942_MOESM1_ESM.doc]

**Supplementary Information for**

Changes to Yucatán Peninsula precipitation associated with salinity and temperature extremes of the Caribbean Sea during the Maya civilization collapse

Henry C. Wu ^1,2^*, Thomas Felis ^1^*, Denis Scholz ^3^, Cyril Giry ^1^, Martin Kölling ^1^, Klaus P. Jochum ^4^, and Sander R. Scheffers ^5,6^

^1^ MARUM - Center for Marine Environmental Sciences, University of Bremen, 28359 Bremen, Germany.

^2^ Now at Leibniz Centre for Tropical Marine Research (ZMT), 28359 Bremen, Germany

^3^ Institute for Geosciences, Johannes Gutenberg University Mainz, 55099 Mainz, Germany.

^4^ Climate Chemistry Department, Max Planck Institute for Chemistry, 55128 Mainz, Germany.

^5^ Marine Ecology Research Centre, Southern Cross University, Lismore, NSW 2480, Australia.

^6^ School of Earth and Environmental Sciences, The University of Queensland, St. Lucia, QLD 4072, Australia.

*Correspondence and requests for materials should be addressed to Wu (henry.wu@leibniz-zmt.de) or Felis (tfelis@marum.de).

**This PDF file includes:**

**Tables S1 to S2**

**Figures S1 to S8**

**Supplementary References**

**Supplementary Tables**

**Table S1. *Orbicella* spp. Sr/Ca-SST calibrations.**

**Atlantic Ocean *Orbicella* spp. Sr/Ca to sea surface temperature calibration relationships from published studies by Ordinary Least Squares (OLS), Reduced Major Axis (RMA), or Weighted Least Squares (WLS) regressions.**

| **Study** | **Sampling Resolution** | **Type of calibration** | **Slope** | **Y-intercept** |
| --- | --- | --- | --- | --- |
| Swart et al., 2002 ^1^ | ~50 yr^-1^ | OLS | -0.047 | 10.16 |
| Smith et al., 2006 ^2^ | ~14 yr^-1^ | OLS | -0.024 | 9.86 |
| Smith et al., 2006 ^2^ | ~14 yr^-1^ | RMA | -0.028 | 9.96 |
| Saenger et al., 2008 ^3^ | ~6 - 32 yr^-1^ | Growth-dependent OLS | -0.092 | 11.82 |
| Kilbourne et al., 2008 ^4^ | ~12 yr^-1^ | No calibration given, used Swart et al., 2002 ^1^ | | |
| Kilbourne et al., 2010 ^5^ | ~14 yr^-1^ | No calibration given, used Swart et al., 2002 ^1^ and Saenger et al., 2008 ^3^ | | |
| DeLong et al., 2011 ^6^ | ~12 yr^-1^ | WLS | -0.027 | 9.89 |
| Flannery and Poore, 2003 ^7^ | ~14 yr^-1^ | RMA | -0.039 | 10.21 |

**Table S2. *Orbicella* spp. δ^18^O-SST calibrations.**

**Atlantic Ocean *Orbicella* spp. δ^18^O to sea surface temperature calibration relationships from published studies by Ordinary Least Squares (OLS) or Reduced Major Axis (RMA) regressions.**

| **Study** | **Sampling Resolution** | **Type of calibration** | **Slope** | **Y-intercept** |
| --- | --- | --- | --- | --- |
| Leder et al., 1996 ^8^ | ~25 - 55 yr^-1^ | OLS | -0.22 | 1.18 |
| Winter et al., 2000 ^9^ | ~24 yr^-1^ | No calibration given, stated identical to Leder et al., 1996 ^8^ | | |
| Watanabe et al., 2001 ^10^ | ~50 yr^-1^ | OLS | -0.19 | 0.75 |
| Watanabe et al., 2002 ^11^ | ~14 yr^-1^ | No calibration given, used Leder et al., 1996 ^8^ | | |
| Gischler and Oschmann, 2005 ^12^ | ~4 - 8 yr^-1^ | No calibration | | |
| Greer and Swart, 2006 ^13^ | ~12 yr^-1^ | No calibration given, used Leder et al., 1996 ^8^ | | |
| Smith et al., 2006 ^2^ | ~14 yr^-1^ | OLS | -0.09 | -1.67 |
| Smith et al., 2006 ^2^ | ~14 yr^-1^ | RMA | -0.10 | -1.24 |
| Kilbourne et al., 2008 ^4^ | ~12 yr^-1^ | No calibration given, used Leder et al., 1996 ^8^ | | |
| Kilbourne et al., 2010 ^5^ | ~14 yr^-1^ | OLS (Final calibration used Leder et al., 1996 ^8^) | -0.13 | -0.6 |
| Kilbourne et al., 2010 ^5^ | ~14 yr^-1^ | RMA (Final calibration used Leder et al., 1996 ^8^) | -0.26 | 2.75 |

**Supplementary Figures**

**
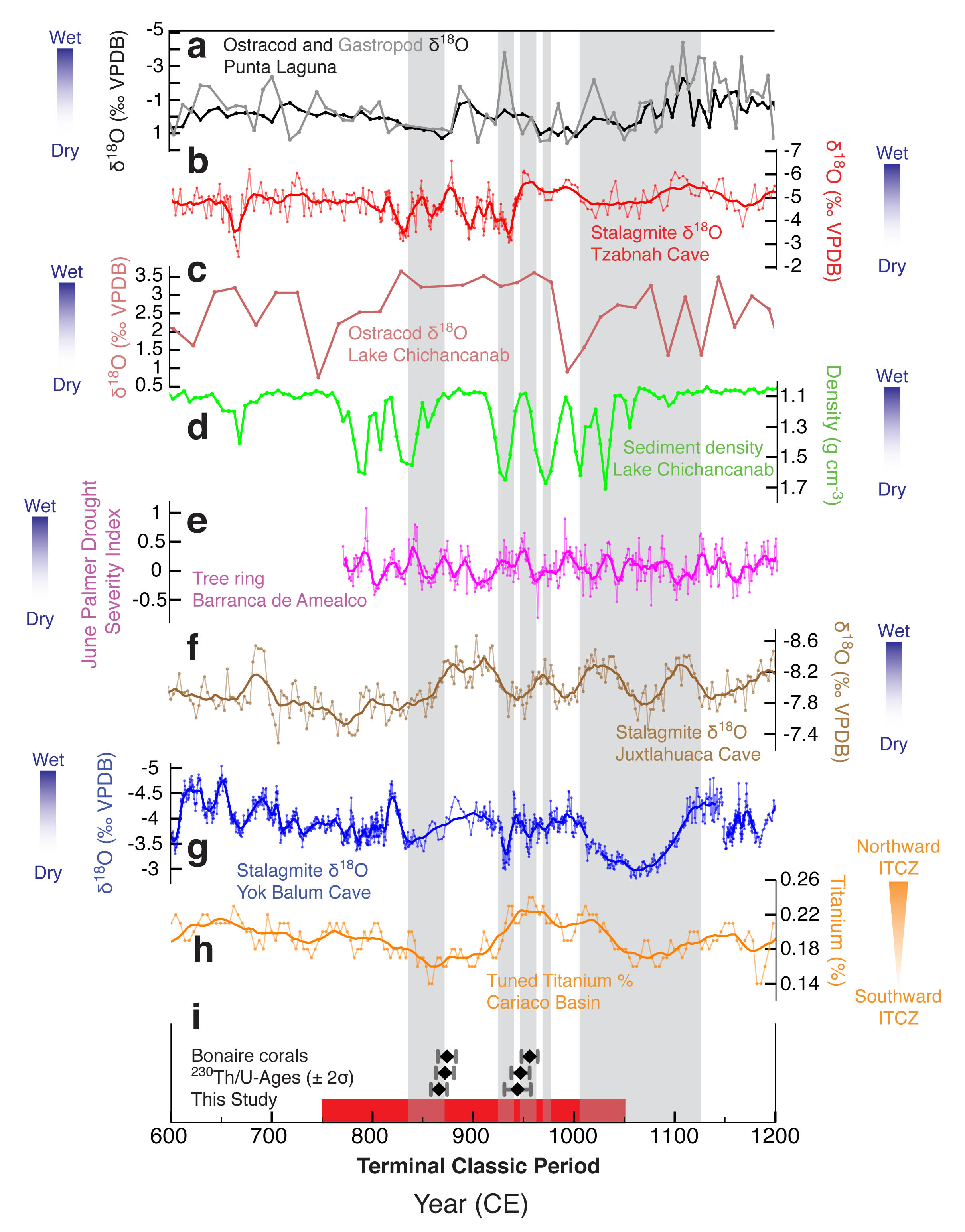
**

**Figure S1. Low-resolution drought records of the Terminal Classic Period.**

Previously published terrestrial summer precipitation records indicating drought conditions over a wide spatial region during the TCP (~CE 750-1050). Furthermore, the records do not depict synchronous temporal drought conditions across this large region. The records are arranged geographically from the north to the south: **(a)** Punta Laguna (ostracod and gastropod δ^18^O)^14^, **(b)** Tzabnah Cave (speleothem δ^18^O)^15^, **(c)** Lake Chichancanab (ostracod δ^18^O)^16^, **(d)** Lake Chichancanab (sediment density)^17^, **(e)** Barranca de Amealco (Palmer Drought Severity Index)^18^, **(f)** Juxlahuaca Cave (speleothem δ^18^O)^19^, **(g)** Yok Balum Cave, Belize (speleothem δ^18^O)^20^, **(h)** Cariaco Basin (tuned sediment titanium % ^20^). **(i)** Fossil coral ^230^Th/U-ages with 2σ-error from this study. The shaded bars demarcate the drier-than-average periods adopted from the well-dated near-annual terrestrial summer precipitation record^20^.

**
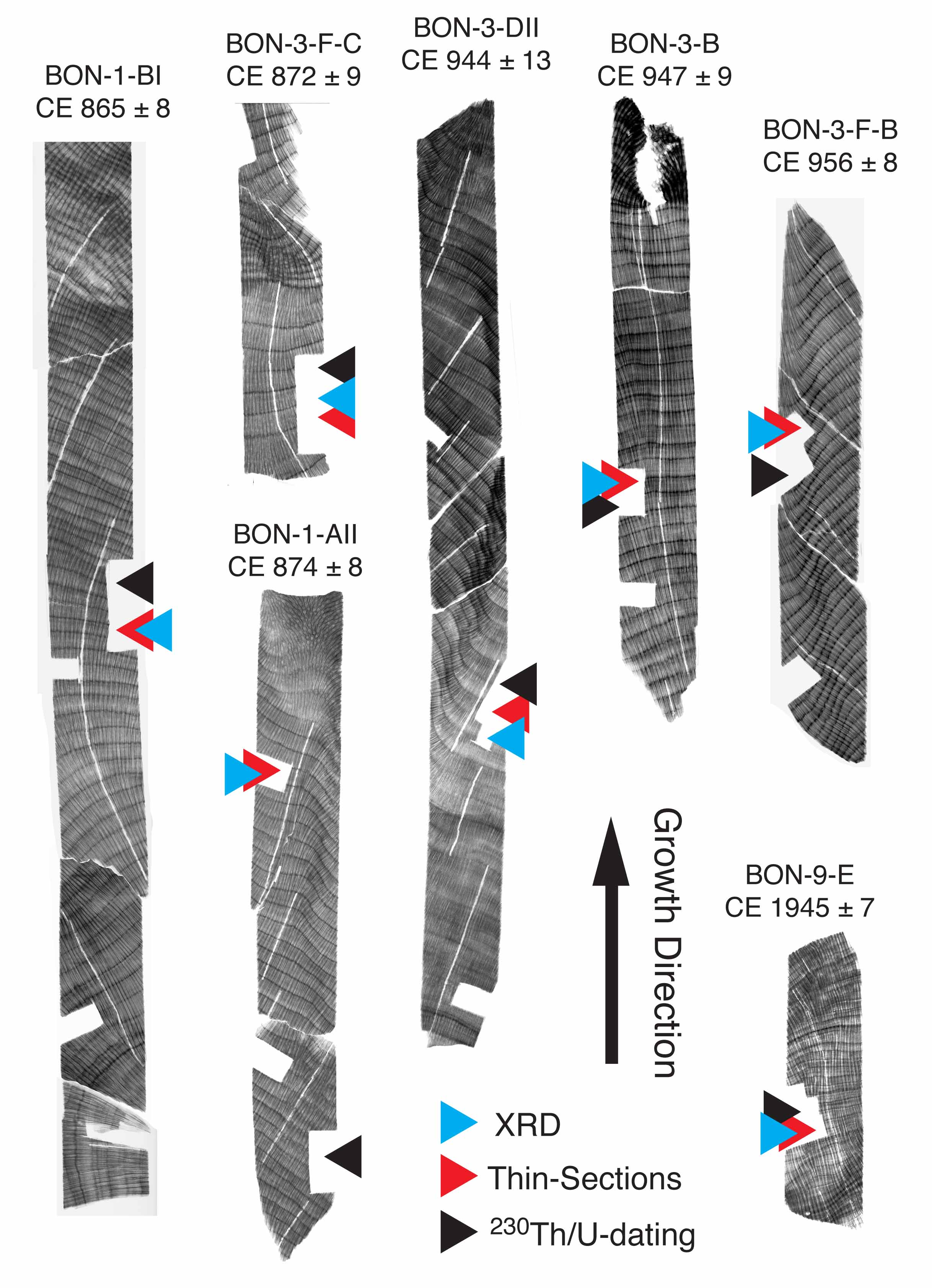
**

**Figure S2. X-radiograph collage of fossil Bonaire corals.**

The coral cores are 6.3 cm in diameter shown here with micro-sampling transects at every millimetre interval down the entire length of available core. All sampling transects are perpendicular to the maximum growth axis. Next to each individual coral colony the sample horizon is marked where material was retrieved for powder XRD (blue triangles, Table S1), ^230^Th/U-dating (black triangles, Table 2), and microstructure petrographic thin sections analyses (red triangles, Fig. S3). ^230^Th/U-ages of each individual colony are shown as ages CE and listed with 2σ-uncertainty.

**

**

**Figure S3.** **Thin sections of fossil Bonaire corals.**

A collection of representative petrographic thin section images of the seven *Orbicella annularis* sensu lato (‘species complex’) corals from Bonaire identified by age in CE. The images display pristine centres of calcification and aragonite skeleton without any incidences of major alteration from diagenesis. In addition, the coral skeletal pore spaces are without infilling between different layers of coral material (thecal walls and dissepiments) by secondary aragonite.

**
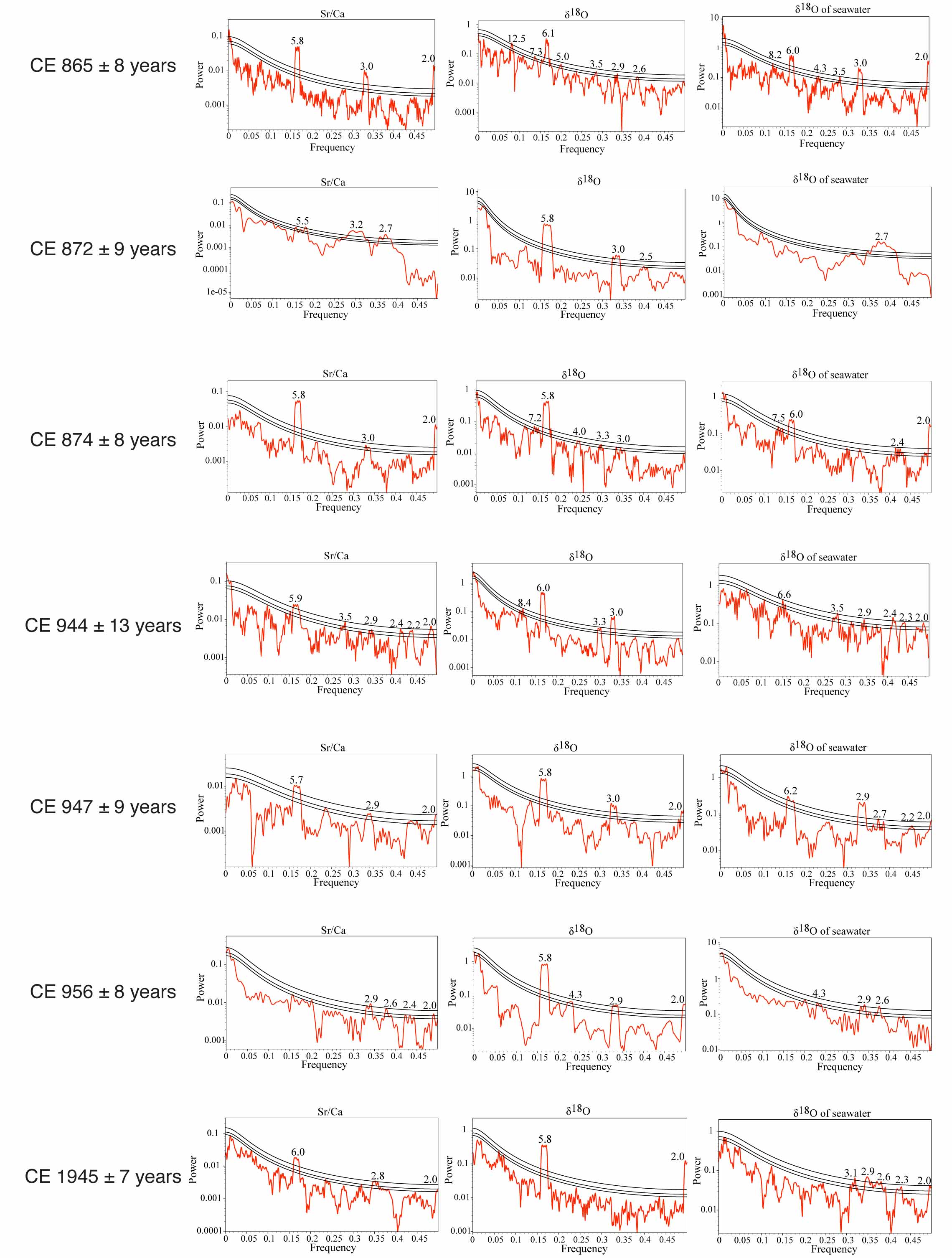
**

**Figure S4.** **Spectral analysis results of fossil Bonaire corals.**

Multi-Taper Method (MTM) spectral analysis and significance plots (red) of normalized and mean seasonal cycles removed records of coral Sr/Ca (left column), δ^18^O (middle column), and δ^18^O_sw_ (right column). Significance was determined relative to a red noise null hypothesis determined with the robust method of noise background estimation^21^ with configuration of 3 for tapers and 2 for resolution^22^. The fossil corals are oriented based on age from oldest (top) to the youngest (bottom). In each individual panel, three black lines demarcate the 99% (top), 95% (middle), and 90% (bottom) significance levels with significant spectral peaks labelled in years.

**
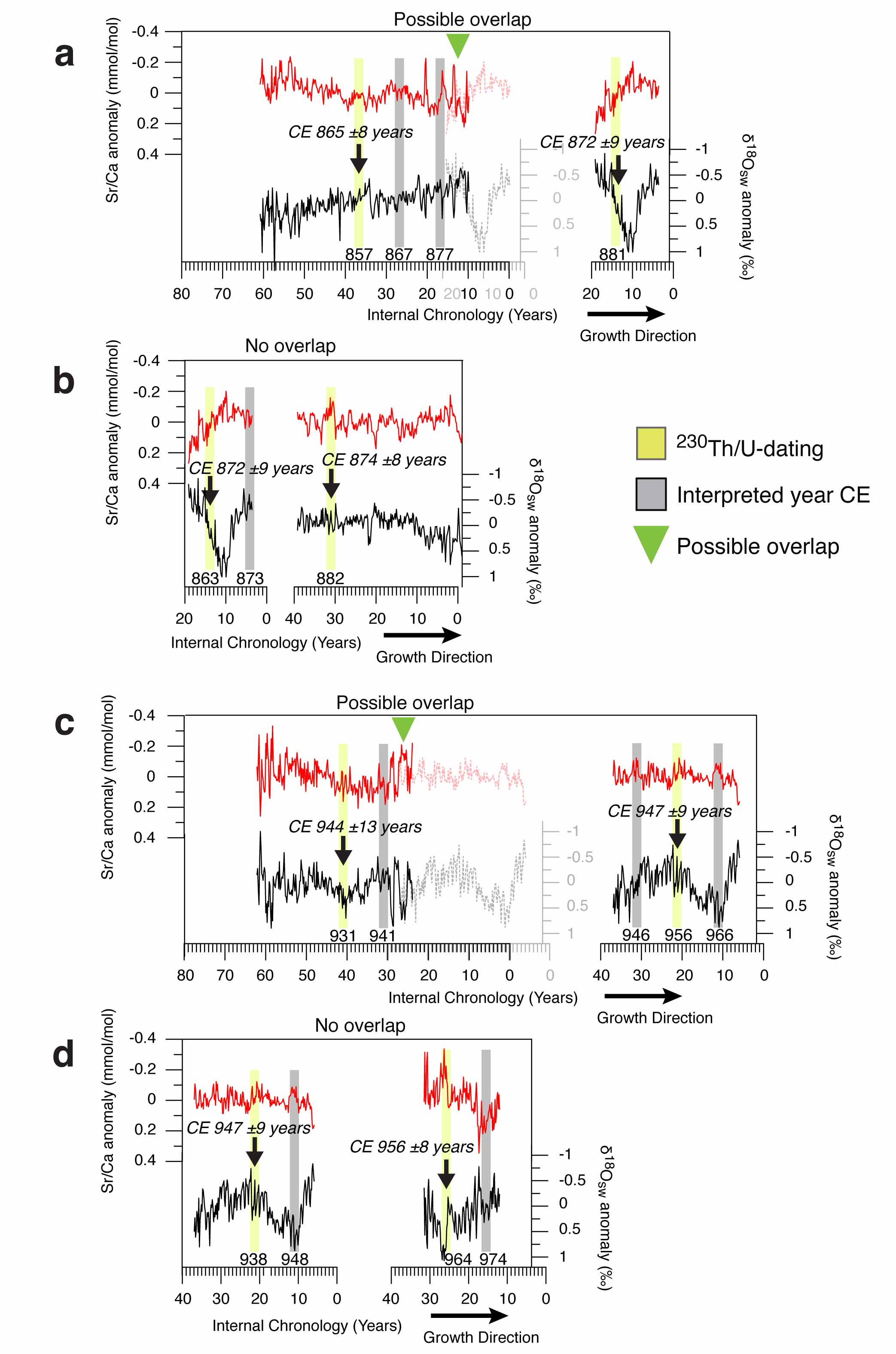

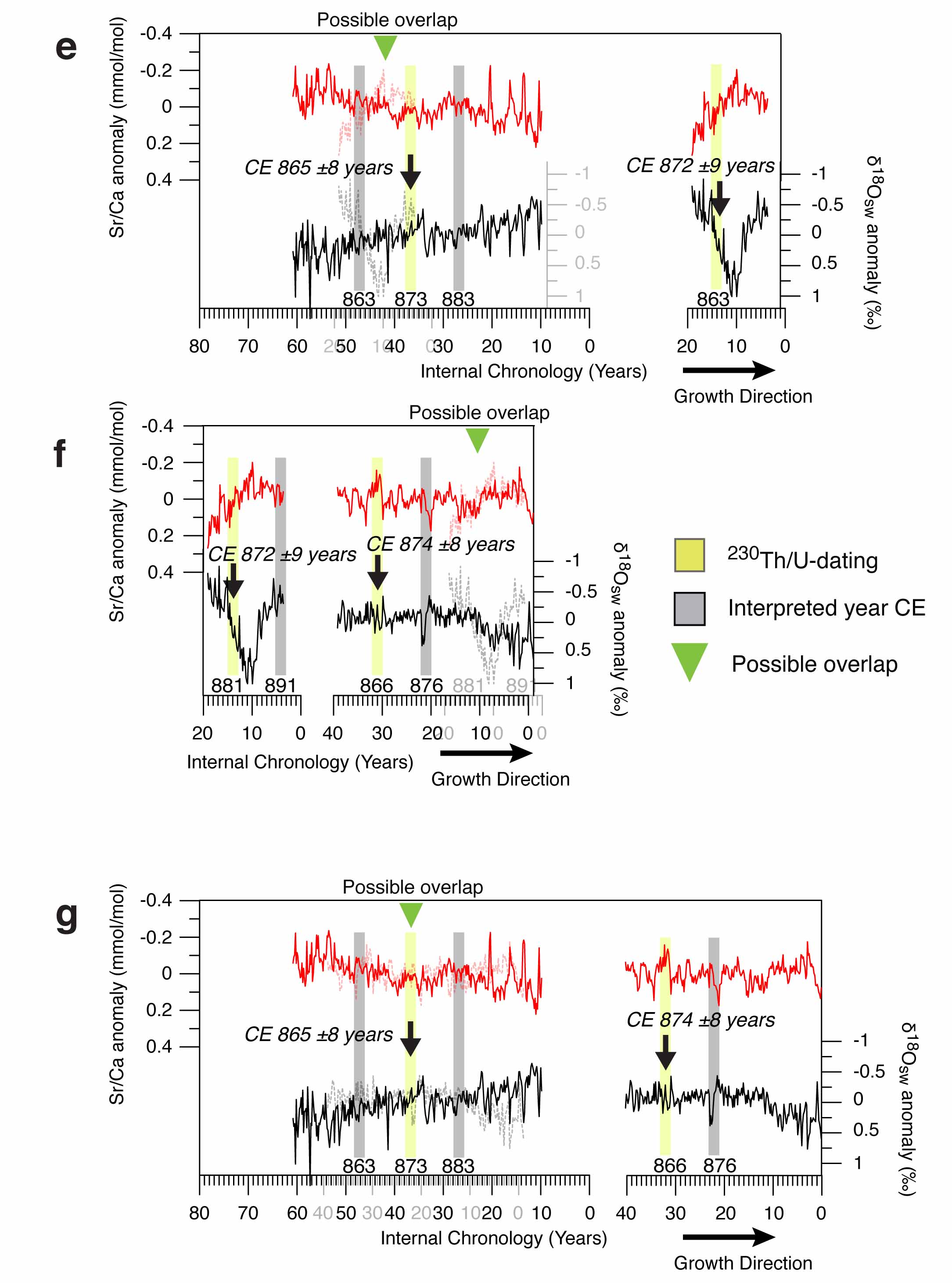

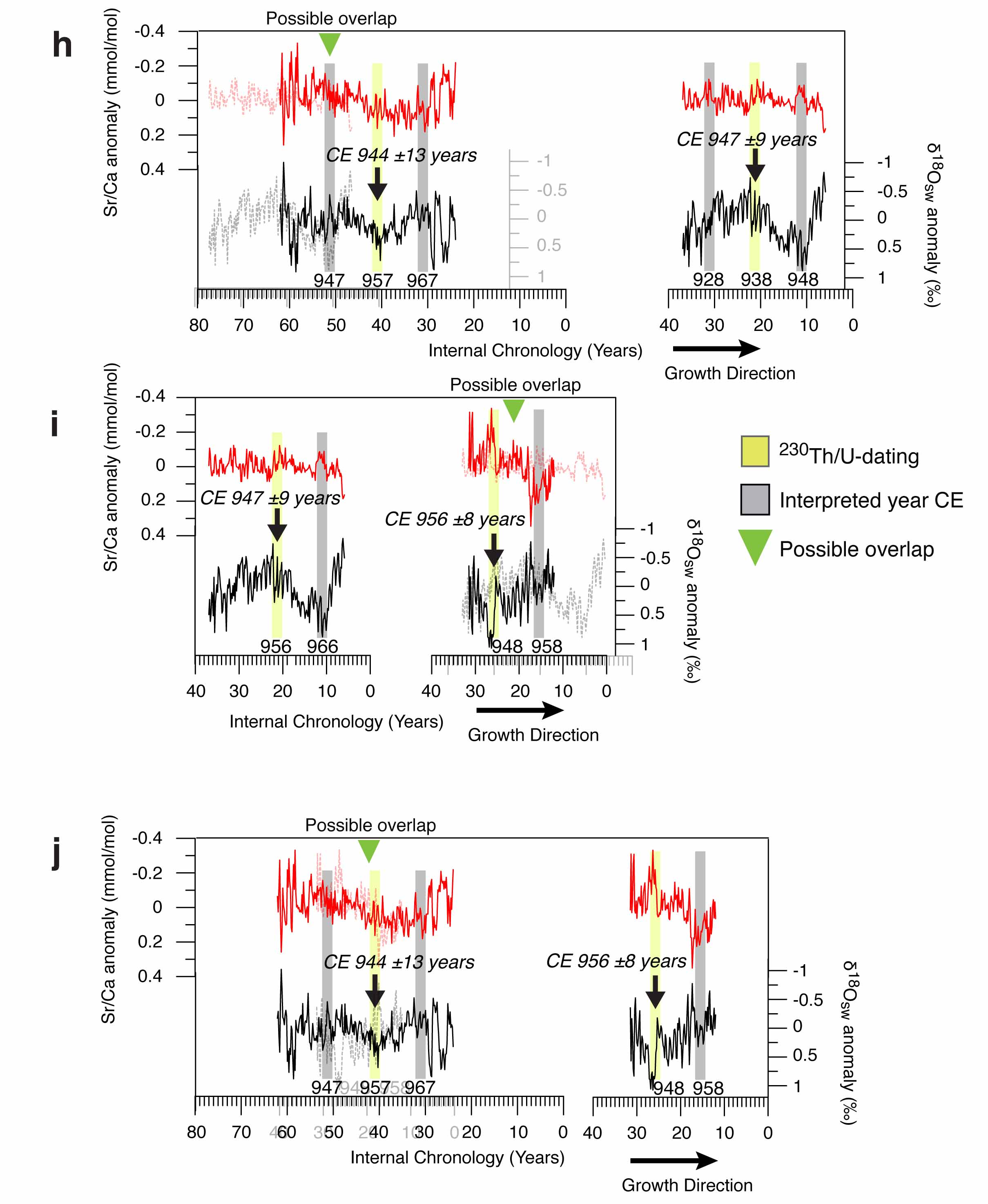
**

**Figure S5.** **Fossil Bonaire coral ^230^Th/U-dating** **adjustments.**

The six individual fossil Bonaire coral samples adjusted towards the extremes within the precision ^230^Th/U-dating method’s 2σ-error uncertainty (Table 2) indicating possible age displacement of the records. Dating uncertainty time windows both as raw (top) and interpreted (bottom) are shown with possible overlap (yellow) based on the location of the ^230^Th/U-dating samples from the coral slab (Fig. S2) and internal stable isotope and trace elements chronology calculations (grey; Table 1). The results show possible ‘merging’ sections between floating individual coral time windows into a singular time-series. Despite the almost perfect matches between some of the coral core samples, we believe the most accurate representation of our results is presented as individual coral time windows that minimizes the inherent age-errors.

**
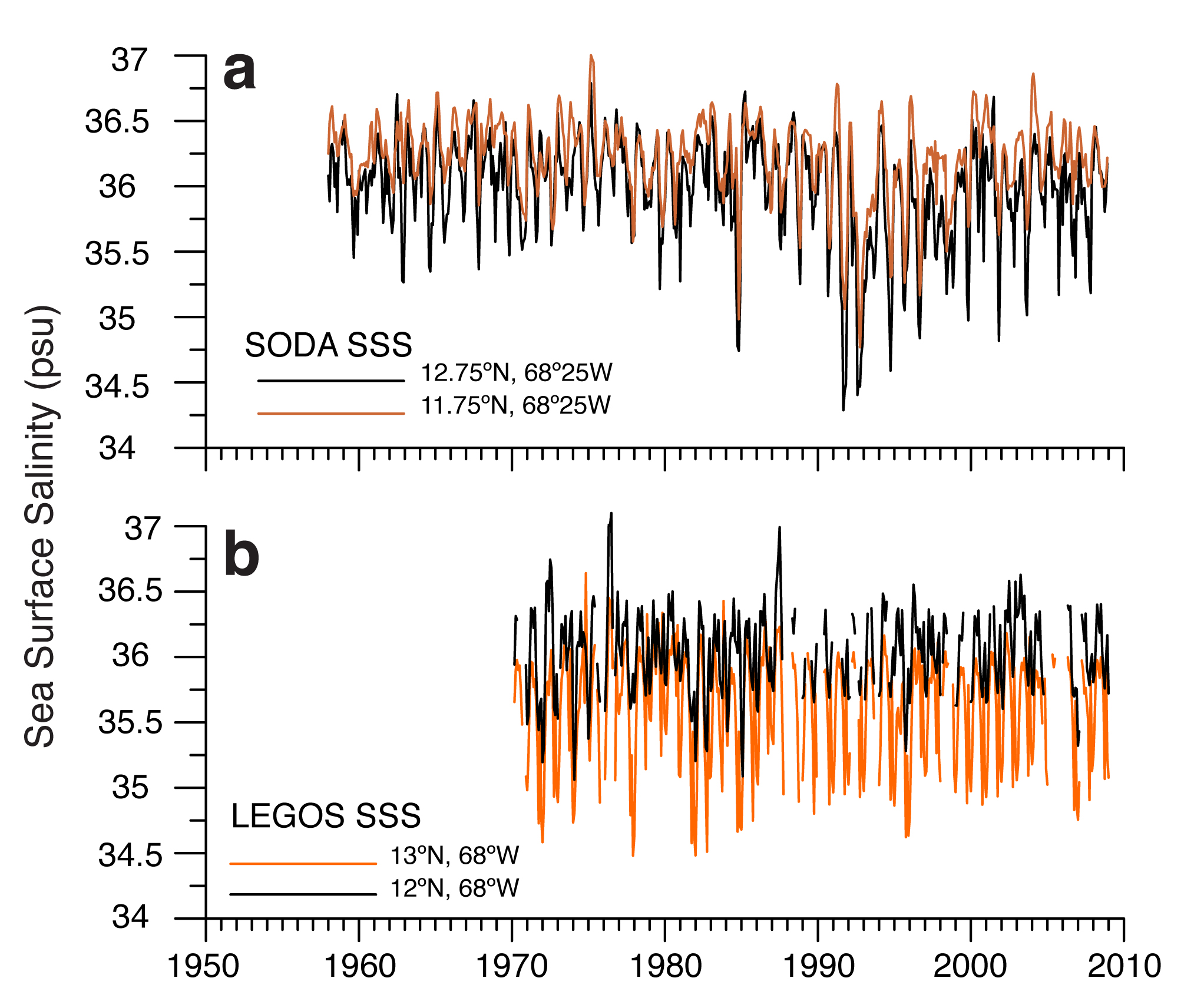
**

**Figure S6.** **Modern instrumental sea surface salinity comparison.**

Gridded instrumental sea surface salinity (SSS) datasets showing the lack of reproducibility over similar grid sizes surrounding Bonaire. **(a)** Simple Ocean Data Assimilation (SODA) ver. 2.1.6 ^23^ for the 1º latitude by 1º longitude grids of 11.75ºN, 68.25ºW (red) and 12.75ºN, 68.25ºW (black). **(b)** The 1º latitude by 1º longitude grids of 12ºN, 68ºW (black) and 13ºN, 68ºW (orange) form the French Environmental Observation Services (IRD), Laboratoire d’Etudes en Géophysique et Océanographie Spatiales (LEGOS)^24^. Differences between these two different datasets can be as large as 1.3 psu (e.g. September 1989), which equates to a 0.26‰ change in reconstructed δ^18^O_sw_ based on the linear SSS-coral δ^18^O_sw_ relationship (0.20‰ per psu)^11^.

**
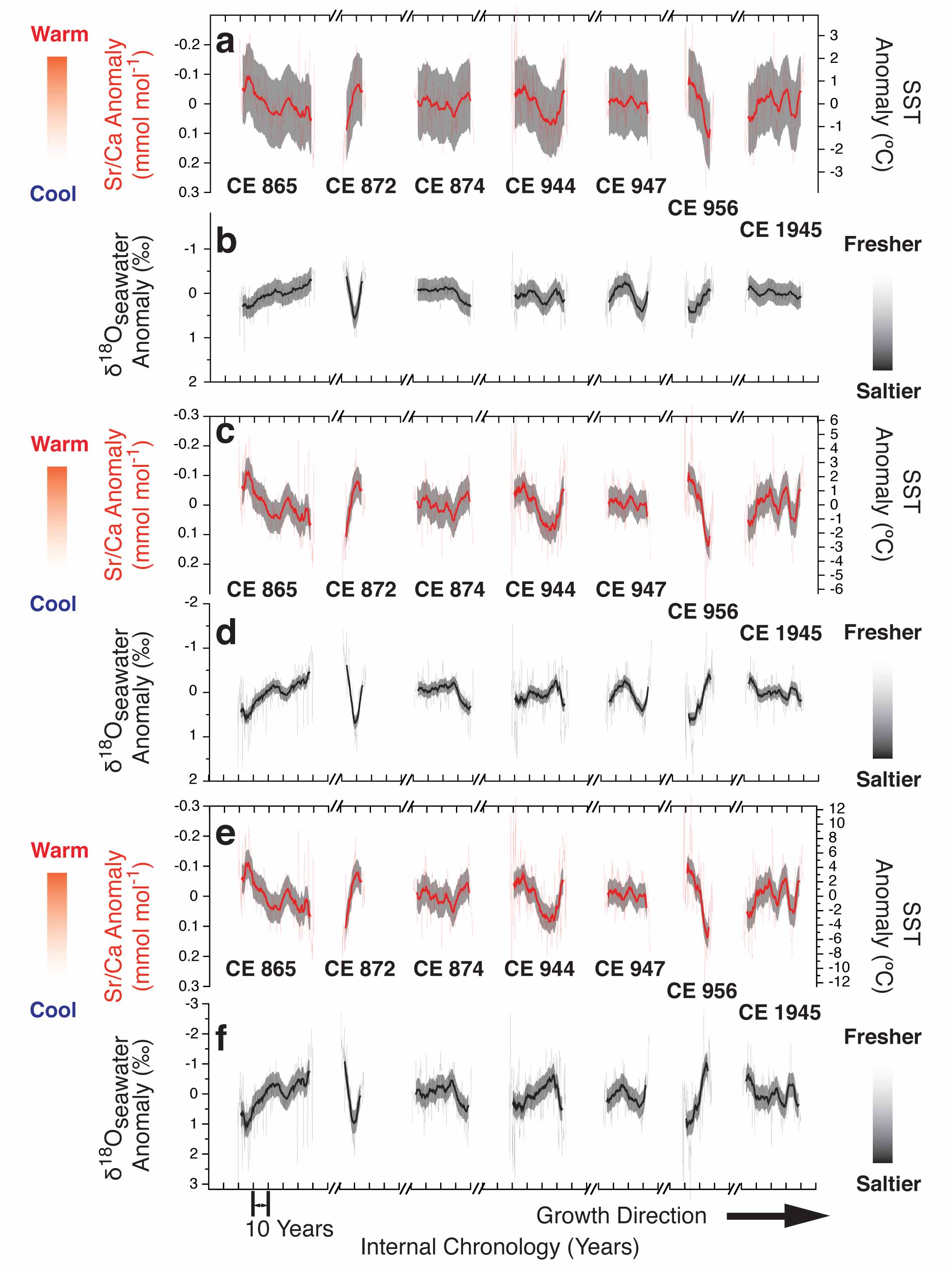
**

**Figure S7. Multi-calibration results of fossil Bonaire corals.**

Sr/Ca anomaly of each coral time-series converted to SST anomaly following the Sr/Ca-SST calibration relationships of *Orbicella* spp. listed in Table S1 including the resulting δ^18^O_sw_ reconstructions. **(a)** The Sr/Ca-SST calibration relationship used in this study^3^ (Figs. 3-4) for the reconstruction of SST anomaly (ºC) and **(b)** the succeeding δ^18^O_sw_ reconstruction. **(c)** The mid amplitude Sr/Ca-SST calibration^1^ and resulting **(d)** δ^18^O_sw_ reconstruction. **(e)** The high amplitude calibration of Sr/Ca-SST^2^ and the resulting **(f)** δ^18^O_sw_ reconstruction. The conventional *Orbicella* spp. δ^18^O-SST transfer function from ref. 8 was used for all δ^18^O_sw_ reconstructions. The grey envelopes surrounding both the Sr/Ca-derived SST anomaly and δ^18^O_sw_ anomaly values in all panels are the composite uncertainty of both analytical (i.e., δ^18^O and Sr/Ca) and regression errors (i.e., δ^18^O-SST and Sr/Ca-SST) as given in the original publication following established error propagation method^25^.

**
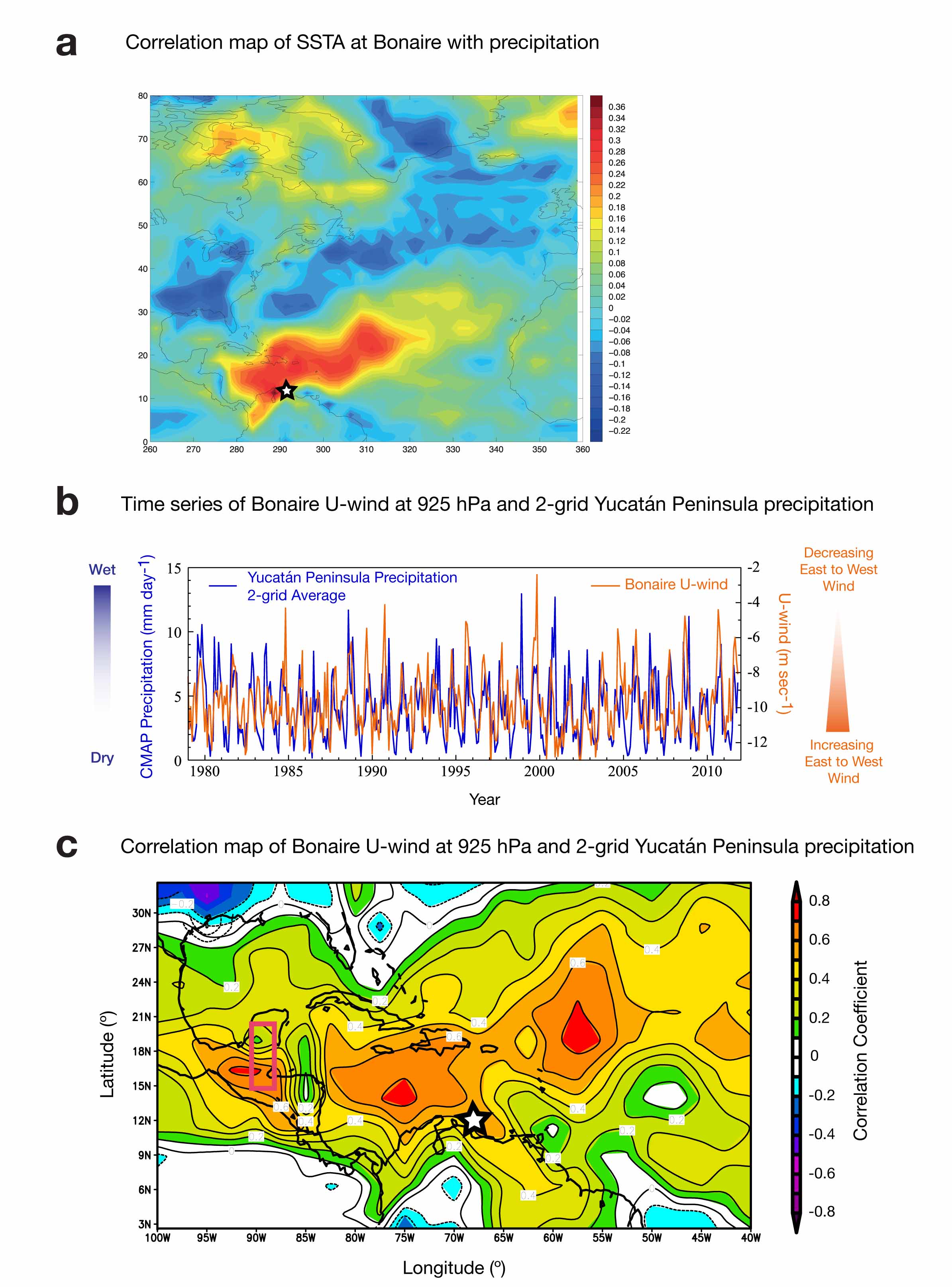
**

**Figure S8. Correlation maps of Caribbean ocean-atmospheric interactions.**

**(a)** Correlation map between annual sea surface temperature anomaly^26^ at Bonaire cantered on 12ºN, 68ºW and NASA Global Precipitation Climatology Project version 2 (GPCP v.2) annual precipitation^27^. **(b)** Time series comparison of Bonaire U-wind at 925 hPa from NCAR Reanalysis^28^ and 2-grid Yucatán Peninsula average precipitation from GPCP v.2^27^ . **(c)** Correlation map of Bonaire U-wind at 925 hPa from NCAR Reanalysis^28^ and 2-grid (grey box) Yucatán Peninsula average precipitation from GPCP v.2^27^ . Correlation maps provided by the NOAA/ESRL Physical Sciences Division, Boulder, Colorado, from their Web site at http://www.esrl.noaa.gov/psd/.

**References for Supplementary Information**

1. Swart, P. K., Elderfield, H. & Greaves, M. J. A high-resolution calibration of Sr/Ca thermometry using the Caribbean coral *Montastraea annularis*. *Geochemistry, Geophys. Geosystems* **3,** 1–11 (2002).

2. Smith, J. M., Quinn, T. M., Helmle, K. P. & Halley, R. B. Reproducibility of geochemical and climatic signals in the Atlantic coral *Montastraea faveolata*. *Paleoceanography* **21,** PA1010 (2006).

3. Saenger, C., Cohen, A. L., Oppo, D. W. & Hubbard, D. Interpreting sea surface temperature from strontium/calcium ratios in *Montastraea* corals: Link with growth rate and implications for proxy reconstructions. *Paleoceanography* **23,** PA3102 (2008).

4. Kilbourne, K. H. *et al.* Paleoclimate proxy perspective on Caribbean climate since the year 1751: Evidence of cooler temperatures and multidecadal variability. *Paleoceanography* **23,** PA3220 (2008).

5. Kilbourne, K. H. *et al.* Coral windows onto seasonal climate variability in the northern Caribbean since 1479. *Geochemistry, Geophys. Geosystems* **11,** n/a-n/a (2010).

6. DeLong, K. L., Flannery, J. A., Maupin, C. R., Poore, R. Z. & Quinn, T. M. A coral Sr/Ca calibration and replication study of two massive corals from the Gulf of Mexico. *Palaeogeogr. Palaeoclimatol. Palaeoecol.* **307,** 117–128 (2011).

7. Flannery, J. A. & Poore, R. Z. Sr/Ca Proxy Sea-Surface Temperature Reconstructions from Modern and Holocene *Montastraea faveolata* Specimens from the Dry Tortugas National Park, Florida, U.S.A. *J. Coast. Res.* **63,** 20–31 (2013).

8. Leder, J. J., Swart, P. K., Szmant, A. M. & Dodge, R. E. The origin of variations in the isotopic record of scleractinian corals: I. Oxygen. *Geochim. Cosmochim. Acta* **60,** 2857–2870 (1996).

9. Winter, A., Ishioroshi, H., Watanabe, T., Oba, T. & Christy, J. Caribbean sea surface temperatures: two-to-three degrees cooler than present during the Little Ice Age. *Geophys. Res. Lett.* **27,** 3365–3368 (2000).

10. Watanabe, T., Winter, A. & Oba, T. Seasonal changes in sea surface temperature and salinity during the Little Ice Age in the Caribbean Sea deduced from Mg/Ca and ^18^O/^16^O ratios in corals. *Mar. Geol.* **173,** 21–35 (2001).

11. Watanabe, T., Winter, A., Oba, T., Anzai, R. & Ishioroshi, H. Evaluation of the fidelity of isotope records as an environmental proxy in the coral *Montastraea*. *Coral Reefs* **21,** 169–178 (2002).

12. Gischler, E. & Oschmann, W. Historical climate variation in Belize (Central America) as recorded in scleractinian coral skeletons. *Palaios* **20,** 159–174 (2005).

13. Greer, L. & Swart, P. K. Decadal cyclicity of regional mid-Holocene precipitation: Evidence from Dominican coral proxies. *Paleoceanography* **21,** PA2020 (2006).

14. Curtis, J., Hodell, D. & Brenner, M. Climate variability on the Yucatan Peninsula (Mexico) during the past 3500 Years, and implications for Maya cultural evolution. *Quat. Res.* **46,** 37–47 (1996).

15. Medina-Elizalde, M. *et al.* High resolution stalagmite climate record from the Yucatán Peninsula spanning the Maya terminal classic period. *Earth Planet. Sci. Lett.* **298,** 255–262 (2010).

16. Hodell, D., Curtis, J. & Brenner, M. Possible role of climate in the collapse of classic maya civilization. *Nature* **375,** 391–394 (1995).

17. Hodell, D. A., Brenner, M. & Curtis, J. H. Terminal Classic drought in the northern Maya lowlands inferred from multiple sediment cores in Lake Chichancanab (Mexico). *Quat. Sci. Rev.* **24,** 1413–1427 (2005).

18. Stahle, D. W. *et al.* Major Mesoamerican droughts of the past millennium. *Geophys. Res. Lett.* **38,** 2–5 (2011).

19. Lachniet, M. S., Bernal, J. P., Asmerom, Y., Polyak, V. & Piperno, D. A 2400 yr Mesoamerican rainfall reconstruction links climate and cultural change. *Geology* **40,** 259–262 (2012).

20. Kennett, D. J. *et al.* Development and disintegration of Maya political systems in response to climate change. *Science* **338,** 788–791 (2012).

21. Mann, M. E. & Lees, J. M. Robust estimation of background noise and signal detection in climatic time series. *Clim. Change* **33,** 409–445 (1996).

22. Ghil, M. *et al.* Advanced spectral methods for climatic time series. *Rev. Geophys.* **40,** 1–41 (2002).

23. Carton, J. A. & Giese, B. S. A reanalysis of ocean Climate using Simple Ocean Data Assimilation (SODA). *Mon. Weather Rev.* **136,** 2999–3017 (2008).

24. Reverdin, G., Kestenare, E., Frankignoul, C. & Delcroix, T. Surface salinity in the Atlantic Ocean (30°S–50°N). *Prog. Oceanogr.* **73,** 311–340 (2007).

25. Nurhati, I. S., Cobb, K. M. & Di Lorenzo, E. Decadal-scale SST and salinity variations in the central tropical Pacific: Signatures of natural and anthropogenic climate change. *J. Clim.* **24,** 3294–3308 (2011).

26. Smith, T. M., Reynolds, R. W., Peterson, T. C. & Lawrimore, J. Improvements to NOAA’s Historical Merged Land–Ocean Surface Temperature Analysis (1880–2006). *J. Clim.* **21,** 2283–2296 (2008).

27. Adler, R. F. *et al.* The Version-2 Global Precipitation Climatology Project (GPCP) Monthly Precipitation Analysis (1979–Present). *J. Hydrometeorol.* **4,** 1147–1167 (2003).

28. Kalnay, E. *et al.* The NCEP/NCAR 40-Year Reanalysis Project. *Bull. Am. Meteorol. Soc.* **77,** 437–471 (1996).
